# MicroRNA155 Expression in Relation to BDCAF Scored Behçet's Disease in an Egyptian Patients' Sample

**DOI:** 10.2174/1874312901812010115

**Published:** 2018-07-31

**Authors:** Sally S. Hassouna, Manal Y. Tayel, Dalal M. ElKaffash, Ahmed M. Abdelhady, Eman H. Elsayed

**Affiliations:** 1 Department of Internal Medicine, Faculty of Medicine, Alexandria University, Alexandria, Egypt; 2 Department of Clinical and Chemical Pathology, Faculty of Medicine, Alexandria University, Alexandria, Egypt; 3 Department of Opthalmology, Faculty of Medicine, Alexandria University, Alexandria, Egypt

**Keywords:** Behçet's disease, MicroRNA155, BDCAF, Egyptian patients, miRNA155, CD40

## Abstract

**Objective:**

To discover the possibility of using microRNA155 (miRNA155) expression level as a biomarker of Behçet's Disease (BD) activity or remission.

**Methods:**

Thirty BD patients’ white blood cells (WBCs) miRNA155 expression was measured and compared to WBCs miRNA155 expression in 15 healthy subjects. Assessment of disease activity was done using Behçet's Disease Current Activity Form (BDCAF).

**Results:**

miRNA155 expression significantly decreases with the increase of BD activity scored by BDCAF.

**Conclusion:**

Increased miRNA155 may be used as a biomarker of BD remission and thus in the disease follow up. There could be a prospect of treating the disease *via* microRNA 155 effect enhancement.

## 1 INTRODUCTION

BD is a chronic multi-systemic disease characterized by oral ulcers more than 3 times in the year with at least two of the following: genital ulcers, cutaneous manifestations, ophthalmic, neurologic, or rheumatologic presentation [1].

Pathogenesis remains unclear. However, both adaptive and innate immunity play a vital role, and multiple cytokines appeared to be engaged in different pathways of pathogenesis leading to tissue damage [2]. A strong established relation between BD and human leukocytic antigen B51 (HLA-B*51) was evident in the last years [3, 4]. And there was an evidence of CD40 and CD40 ligands on peripheral blood lymphocytes and L- selectin expression on leucocytes of patients with BD [5, 6]. Some biochemical markers were observed to correlate with BD activity for example, the level of Serum Amyloid A (SAA) which increase with the flares of ocular disease, neurological dysfunction, and the presence of oral ulcers, also it was suggested that increased levels of SAA might denote a thrombotic risk in the disease [7]. Elevated cytokine levels of interleukin 6 (IL-6), interleukin 18 (IL-18), interleukin 8 (IL-8), and interferon α2a (IFN-α2a), and low CXCL11 levels were shown in BD. Also, it was observed that a signature of IL-6, and Tumor Necrosis Factor-α (TNF α) and T helper 17 (Th17) are present as well. This could contribute to uncover the role of cytokines in the appearance of specific clinical features of BD [2].

MiRNAs are endogenous small naturally occurring double stranded (about 21-25 nucleotides) RNAs and were first described by Lee and colleagues in 1993 and were termed microRNAs in 2001. They play important regulatory roles in animals and plants through targeting mRNAs for suppression of translation or cleavage. Researchers have begun to understand that miRNAs influence the output of many protein-coding genes and are considered one of the gene regulatory molecules [8].

MicroRNAs exhibit a variable regulatory function in cell growth and differentiation, and are also associated with different human diseases. Previous research on miRNAs has revealed a new paradigm of gene regulations and pathways involved in the pathogenesis of autoimmune disorders, malignant and heart diseases [9]. Thus human miRNAs are likely to be highly useful with a great promise as biomarkers in diseases’ diagnoses, and are targets for disease intervention and attractive for applying new therapies.

## 2 AIM OF THE STUDY

To show the correlation of miRNA155 expression with BD activity scored using BDCAF and the possibility of its usage as a disease activity or remission marker.

## 3 SUBJECTS

Thirty-three BD patients fulfilling the International Study Group diagnostic Criteria of BD [10] were examined.

Seventeen patients had ocular involvement and 16 with non-ocular involvement were recruited from Alexandria university hospital, and out-patient clinics in a cross sectional pattern. Patients with a history of another auto-inflammatory or autoimmune disease, other form of vasculitis, hypercoagulable state, malignancy, chemotherapy, radiotherapy or long standing diabetes were excluded. An informed consent was taken from each patient. Three of the non-ocular diseased patients’ samples were excluded afterwards for technical errors.

Thirteen BD patients were having positive HLAB51, 18 patients were ANA positive and 7 had positive family history of BD.

Fifteen healthy subjects matched to the patients by age and sex served as the control for miRNA155 expression. Age of the patients ranged between 20 to 55 years old with a mean ± SD of 38.03± 9.94 years, and median of 36.5 years. No significant difference between the patients and control subjects. This was applied also for the sex.

Disease activity has been measured using “BDCAF” score depending on the presence or absence of mouth ulcers, genital ulcers erythema nodosum, pustules, arthralgia, arthritis, diarrhea, nausea/ vomiting, headaches, eye inflammation, new central nervous system involvement and new major vessel inflammation, (over the last four weeks before the visit) and accordingly the patients were divided into two groups; a higher disease activity group with a score equals to or more than 4 out of 12, and a lower disease activity group with a score less than 4. Patients included: are shown in Table **1**.

## 4 METHODOLOGY

miRNA155 detection was estimated by TaqMan® applied biosystems through a serial of events [11-13]. Approval of Ethics Committee was obtained from the Local Ethics Committee of Alexandria University, with a serial number 020850, Chairperson: Dr. Maha Ghanem, IRB No. 0007555-FWA, No. 0018699, on 29-6-2016.

## 5 RESULTS

Our study results showed:

MiRNA155 in the group of highly active BD patients as scored by BDCAF ranged between 0.01 to 1.76 with a mean ± SD = 0.48 ± 0.53 and a median of 0.38, while in the lower activity group ranged between 0.01 to 6.56 with a mean ± SD = 2.08 ± 2.43 and a median of 0.73 and its expression in the control group ranged between 0.11 to 4.8 with a mean= 1.52 ± 1.27 and a median of 1.04. There was a significant difference between the higher activity patients and control group (*p*= 0.032), while there was no significant difference between lower activity patients and control group nor between higher activity and lower activity patients’ groups; *p* values were 1.00 and 0.127 respectively. There was no significant difference in miRNA155 expression in the whole patients group with a range extending from 0.01 – 6.56 with a mean ± SD = 1.28 ± 1.91 and a median 0.44 and control subject where (*p* = 0.075), the comparison is shown in Fig. (**1**).

Correlation between miRNA155 with BDCAF activity score in BD patients; a significant negative correlation between BDCAF score and miRNA155 expression level in BD patients involved in the study, with rs = -0.389, *p* = 0.034, this relation is shown in Fig. (**2**).

## 6 DISCUSSION

BD is considered a serious syndrome that can lead to severe morbidity and sometimes mortality and unfortunately with no dependent biomarkers to assess activity or remission.

The study was made to show the relation between miRNA155 expression and disease activity.

The results showed a significant inversely proportional relation between miRNA155 expression in the WBCs and disease activity and so the increase of miRNA155 level denotes a disease remission.

This can be explained by knowing that several studies presented miR-155 as a major player in Dendritic Cell (DC) and B cell function and responses as what was discovered in [14-16]. Two researches were conducted on mice lacking miR-155 showing aberrant B and T cell functions and defect in antigen presenting cells [17, 18].

Also, the increased miRNA155 expression in disease remission may be owing to the fact mentioned in Martinez-Nunez *et al*, 2011 study [19] about miRNA 155 implication in the inflammatory pro-Th1/M1 and that miRNA155 may be regulating the macrophages M1/M2 balance by modulation of IL13 effects.

It was said that there is an effect E26 transformation-specific-1 (Ets-1) on increased miRNA155 as Quinn *et al*., 2014 [20] said, and that Ets-1 is known to be a suppressor of T helper 17 suppressing the release of pathogenic interleukin 17 (IL17) as appeared in Moisan *et al*,. 2007 study [21] IL-17 is increased in BD patients and there is a further increase with activity, ocular, and neurological involvement as what was said in Lopalco 2017 *et al*. [22], and Dina *et al*., 2015 [23].

Although our study included a more wide disease clinical spectrum, this finding of decreased miRNA155 with increased disease activity was shown before in ocular BD through Zhou *et al*., 2012 [24] which stated that miRNA155 expression levels in PBMCs and DCs was decreased in BD patients suffering from active uveitis and overexpression of miRNA155 in DCs increased the inhibitory interleukin 10 (Il 10) and decreased Il6 and Interleukin 1B (Il1B) expression which are pro-inflammatory cytokines which may contribute to disease flare. it was shown in some studies, such as Lopalco *et al*., 2015, that there is an important role Il6 in BD pathogenesis [2].

Some other studies showed an opposite result to ours and that miRNA155 expression is increased in BD activity, as the study of Tili *et al*., 2007 which found that miR-155 is a potent pro-inflammatory microRNA and stated that Eμ-transgenic miR-155 mice have excessive uncontrolled inflammatory responses, giving excessive Tumor Necrosis Factor Alpha (TNFα) and septic shock hypersensitivity [25].

Other researches showed that miR-155 targeting Ets-1 regulates Th17 response and suppression of miR-155 decreases pathogenic IL-17-expressing T cells amount as in Na *et al*., 2016 study [26].

Also, Ryan *et al*. 2011 study found that miRNA155 fosters autoimmunity through inflammatory T cells development [27]. Also, it was mentioned in Rajasaki *et al*. 2017 that Resolvin D1, through inhibiting miRNA155, decreases experimental corneal immunopathology inflammation [28]. And in Zheng *et al*. 2012 and Chinenov *et al*., 2014, it was shown that glucocorticoids are effective in inflammation termination, mediated by miR-155 expression inhibition in a nuclear factor kappa-light-chain-enhancer of activated B cells and glucocorticoids receptor dependent way [29, 30].

The findings in some studies are consistent with ours and some are not, this is may be owing to different ethnicities, different disease activity in the patients in one study from another, also some researches which had the same results of ours or the opposite were depending only on animal studies and we should take this into consideration. Also, BDCAF scoring is almost subjective, depending on patients’ own words in many instances and this may be not so much accurate.

And thus miRNA155 role in BD pathogenesis is still controversial.

## CONCLUSION

Studies on miRNA155 expression levels in active BD disease should be done on a large base to know if there is a cut off to say the disease is active or in remission and for confirmation of the possibility of its usage as a biomarker for follow up of the patient, and to do studies for Ets-1 enhancement or other means of miRNA155 promotion to see the effect on disease progression and potential of this for disease treatment.

## Figures and Tables

**Fig. (1):**
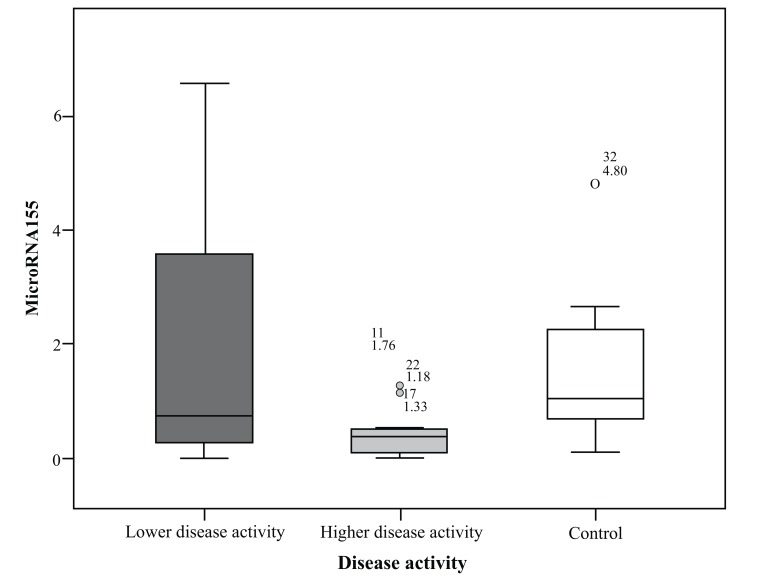
Comparison between miRNA155 expression in the higher disease activity, lower disease activity and control groups.

**Fig. (2):**
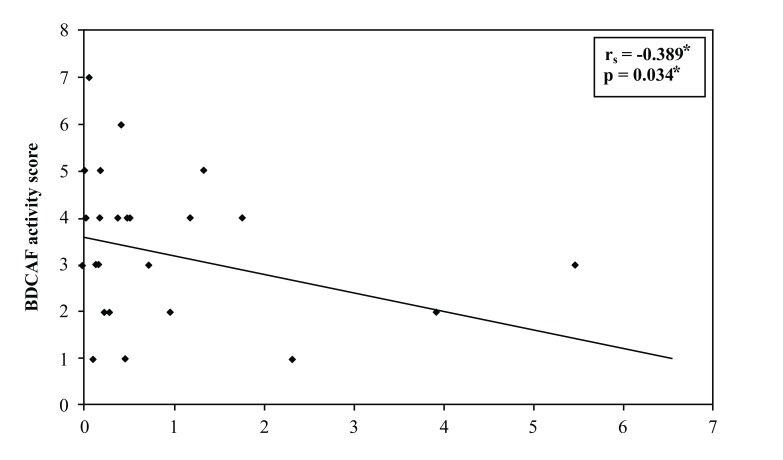
Correlation between miRNA155 expression in BD patients with activity of the disease scored using BDCAF.

**Figure:**
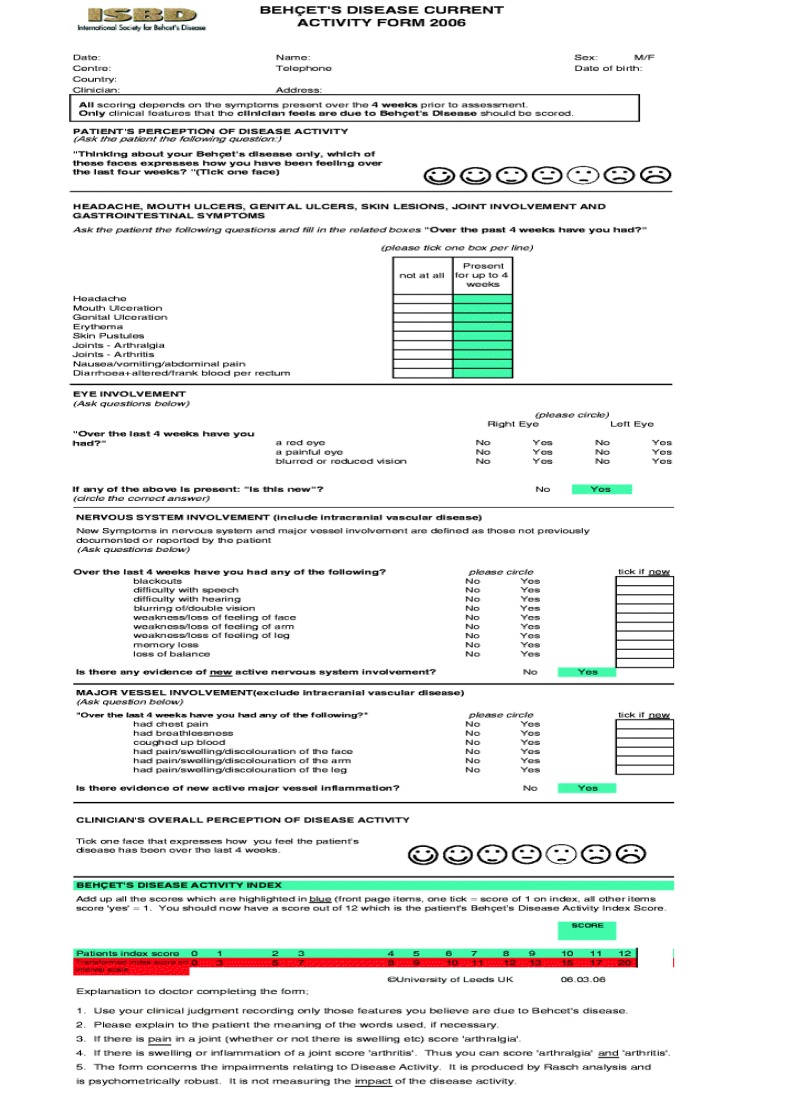


**Table 1:** Clinical Manifestations in Patients that were Active Through the Four weeks Preceding the Study According to BDCAF. (Each of the Mentioned Items Counts for 1 Point as a Score of Disease Activity).

**Active clinical manifestations**	**No.**
Headache	13
Mouth ulceration	11
Genital ulceration	9
Erythema nodosum	7
Skin Pustules	6
Joints – Arthralgia	15
Joints – Arthritis	6
Nausea/Vomiting/Abdominal pain	1
Diarrhea + altered/frank blood per rectum	0
New active eye involvement	3
New active neurological involvement	11
New extra-cranial major vessel involvement	1

Note that some patients had histories of some of these manifestations but were inactive during the study.
